# Incidence and Cost of Pneumonia in Older Adults with COPD in the United States

**DOI:** 10.1371/journal.pone.0075887

**Published:** 2013-10-09

**Authors:** Marian Ryan, Jose A. Suaya, John D. Chapman, William B. Stason, Donald S. Shepard, Cindy Parks Thomas

**Affiliations:** 1 Brandeis University, Schneider Institute on Healthcare Systems, Heller School, Waltham, Massachusetts, United States of America; 2 GlaxoSmithKline Vaccines, Philadelphia, Pennsylvania, United States of America; Maastricht University Medical Center (MUMC), Netherlands

## Abstract

**Objectives:**

To estimate the incidence of pneumonia by COPD status and the excess cost of inpatient primary pneumonia in elders with COPD.

**Study Design:**

A retrospective, longitudinal study using claims linked to eligibility/demographic data for a 5% sample of fee-for-service Medicare beneficiaries from 2005 through 2007.

**Methods:**

Incidence rates of pneumonia were calculated for elders with and without COPD and for elders with COPD and coexistent congestive heart failure (CHF). Propensity-score matching with multivariate generalized linear regression was used to estimate the excess direct medical cost of inpatient primary pneumonia in elders with COPD as compared with elders with COPD but without a pneumonia hospitalization.

**Results:**

Elders with COPD had nearly six-times the incidence of pneumonia compared with elders without COPD (167.6/1000 person-years versus 29.5/1000 person-years; RR=5.7, p <0 .01); RR increased to 8.1 for elders with COPD and CHF compared with elders without COPD. The incidence of inpatient primary pneumonia among elders with COPD was 54.2/1000 person-years compared with 7/1000 person-years for elders without COPD; RR=7.7, p<0.01); RR increased to 11.0 for elders with COPD and CHF compared with elders without COPD. The one-year excess direct medical cost of inpatient pneumonia in COPD patients was **$ 22,697** ($45,456 in cases vs. $ 22,759 in controls (p <0.01)); 70.2% of this cost was accrued during the quarter of the index hospitalization. During months 13 through 24 following the index hospitalization, the excess direct medical cost was **$ 5,941** ($23,215 in cases vs. $ 17,274 in controls, p<0.01).

**Conclusions:**

Pneumonia occurs more frequently in elders with COPD than without COPD. The excess direct medical cost in elders with inpatient pneumonia extends up to 24 months following the index hospitalization and represents $28,638 in 2010 dollars.

## Introduction

Chronic obstructive pulmonary disease (COPD), including emphysema and chronic bronchitis, is the fourth leading cause of death among persons age 65 years and above in the U.S. COPD in the elderly results in an increased risk of hospital admission for infectious disease such as pneumonia, increased morbidity and mortality, and increased health care costs[1,2]. In 2007 the National Heart Lung Blood Institute (NHLBI) estimated the total cost of COPD in the U.S. at $42.6 billion, of which $26.7 billion was direct medical costs[3]. 

Individuals with COPD experience one to three exacerbations a year with some going undiagnosed and the most severe requiring hospitalization[4,5]. Acute exacerbations in those with COPD are caused by upper respiratory tract infections, environmental exposures, and lower respiratory tract infections such as pneumonia. COPD is a disease characterized by frequent acute exacerbations, progressive lung impairment, and upper and lower respiratory tract infections including pneumonia[6]. Importantly, older persons with chronic respiratory diseases such as COPD are at increased risk of contracting pneumonia and associated death[1,7–9]. 

Acute exacerbations due to pneumonia that result in hospitalizations and readmissions are the major contributors to the economic burden of COPD[10]. As such, COPD results in considerable morbidity leading to decreased quality of life and increased health care cost. Annual Medicare payments for beneficiaries diagnosed with COPD have been estimated at $21,409; a cost second only to Medicare payments for beneficiaries with Chronic Kidney Disease[11]. Moreover, studies have found that congestive heart failure (CHF) co-occurs frequently in COPD patients, shares the same risk factors as COPD, increases the risk of mortality in elders with COPD[12–16], and is also a high cost chronic condition with estimated annual Medicare payments of $20,545 per beneficiary[11]. Independently, CHF increases the risk of hospitalization for pneumonia[17].

Although the incidence and cost of pneumonia in the elderly has been documented, less is known about the incidence and economic impact of pneumonia in elderly persons with underlying COPD. Pneumonia is an observable and potentially preventable cause of COPD acute exacerbation in elders. Our study builds upon an earlier study of Medicare beneficiaries[18] to estimate: (1) the crude incidence rates of pneumonia by COPD status; and (2) the excess direct medical cost of inpatient primary pneumonia in COPD patients. 

## Methods

### Data Sources

The Centers for Medicare & Medicaid Services (CMS) Chronic Condition Warehouse (CCW) data [19] were used for these analyses. The CCW is a 5% random sample of nearly 1.6 million fee-for-service (FFS) Medicare beneficiaries aged 65 and older and continuously enrolled in Medicare Parts A and B during the study years of 2004-2007, or until death. Medicare enrollment and claims data for 2005-2007 were linked so that each sampled beneficiary who had been continuously enrolled during these three years or until death was included in the person-level analytical file. Therefore, analyses used linked data for years 2005 through 2007. Specifically, to permit the necessary linkages across years, we obtained research identifiable files (RIF), through a data use agreement (DUA). To ensure the protection of beneficiaries' privacy, the DUA was reviewed by the CMS Privacy Board, the data were maintained securely with access limited to authorized persons, and the study was approved by the Brandeis University Institutional Review Board (IRB) and its HIPAA board as well as the Centers for Medicare & Medicaid Services. As the investigators had no contact with beneficiaries, did not have their names, addresses, or Social Security numbers, and report only aggregate information, the IRB agreed that informed consent from study participants was not necessary. 

Descriptive statistics on the sample population were based on year 2004 data; 2004 data also provided the 12-month look-back period. Medicare beneficiaries enrolled in health maintenance organizations were excluded because claims data were incomplete. Additional information on the CCW can be found in File S1. 

### Study Cohorts

Two patient cohorts and a sub-cohort were specified using the linked Medicare beneficiary files. The initial cohort of interest was patients with COPD observed either in past history or during the study period. Any Medicare beneficiaries not identified with COPD were put into the non-COPD patient cohort. Within the patient cohort of elders identified with COPD, concomitant CHF was identified and these patients were put into the COPD and CHF patient cohort.

### Classification of COPD Status

The presence or absence of COPD was determined by using the claims-based ‘flags’ for COPD in the CCW Beneficiary Summary File A COPD ‘flag’ was generated by CCW if any of the following ICD-9 codes (491.0-2, 491.8,9, 492.0, 492.8, 494.0, 494.1, and 496) were observed during a one-year period in at least one inpatient, skilled nursing home care, or home health agency claim, or two Part B claims, including any combination of claims at least one day apart^8^. The CCW summary files denoted COPD in beneficiary history beginning in 1999; we observed COPD during the study period by using the COPD ‘flag’ that contained the date of ‘first-observed” COPD.

### Identification of Coexisting CHF

Within individuals with observed COPD the coexistence of CHF was identified by using the CCW ‘flag’ for CHF. The CHF ‘flag’ was generated if any of the following ICD-9 codes (398.91, 402.1, 402.11, 402.91, 404.01, 404.11, 404.91, 404.03, 404.13, 404.93, 428.0, 428.1, 428.20, 428.21-.23, 428.30-.33, 428.40-.43, 428.9) were observed during a one-year period in at least one inpatient, skilled nursing home care, home health agency claim, or Part B claims^8^. 

### Episodes of Pneumonia

Pneumonia episodes were identified for the entire sampled population using the linked Medicare claims files from January 1, 2005 through December 31, 2007. Pneumonia was defined as any evaluation and management (E&M) service containing ICD-9 codes 480-483, 485-487.0, and 038.2, a tested algorithm for the identification of pneumonia^14^. Beginning with the date of the first pneumonia claim and looking forward 60 days for evidence of a hospitalization, initial stratification was made into pneumonia with hospitalization versus pneumonia observed only in the outpatient setting. Hospitalized pneumonia was stratified subsequently into pneumonia as the primary or principal diagnosis (inpatient primary pneumonia) versus pneumonia coded as a secondary diagnosis. New pneumonia episodes over the study period were defined and stratified in the same manner with any new pneumonia episode preceded by a 60-day “clean” period of no service claims for a pneumonia diagnosis. Crude incidences rates for pneumonia overall (any pneumonia) and by type (outpatient or inpatient primary pneumonia) were calculated in COPD and in non-COPD persons. In addition, relative rates were calculated to compare pneumonia incidence rates across cohorts of elders based on ICD-9 codes—persons without COPD, those with COPD and persons with COPD and CHF (denoted below as COPD/CHF).

### Excess Direct Medical Cost of Inpatient Primary Pneumonia in Persons with COPD

Direct medical costs were calculated using “Medicare-allowable-charges”; the sum of Medicare spending including, other third party payments, and beneficiary out-of-pocket payments. These are considered a good proxy for cost. Outpatient pharmacy costs were not included because Part D Medicare data were not available. Subsequently, excess direct medical cost was estimated using propensity-score matching to compare direct medical care costs of cases (persons with COPD and hospitalized in 2005 with primary pneumonia) with controls ( ‘matched ‘ persons with COPD but not hospitalized with pneumonia). Selected controls matched the case exactly on: age (year of birth ± two years), gender, Medicare initial eligibility status (disabled or end stage renal disease [ESRD] versus only age 65 or over), history of CHF or asthma or lung cancer, inpatient stay or a skilled nursing facility stay during the prior 90 days, and category of Medicare allowable charges during the prior 90 days (zero, below the median, between median and 75^th^ percentile and above 75^th^ percentile). One control was selected for each case from among the potential controls using propensity score matching, via the nearest neighbor technique, in which the one best match is chosen for each case without replacement. Both cases and potential controls had a diagnosis of COPD, had no evidence of pneumonia in the preceding 12 months, and were enrolled in Medicare Parts A and Part B for at least one year prior to the index pneumonia hospitalization (or matched month of controls) and for the subsequent two years or until death. 

Propensity scores were generated by estimating the conditional probability of being hospitalized for pneumonia, given measured covariates. These covariates, selected because they might otherwise confound the relationship between pneumonia and cost[8,9,20–25], included: the quarter in which the index pneumonia hospitalization occurred; age, gender, race, a low-income variable (the bottom quartile for median household income assigned by zip code), initial Medicare status (ESRD/disabled versus aged), 37 comorbid conditions found to be associated with pneumonia[24] that were present in the past three months and past 12 months; the total number of chronic conditions (linear and squared); a hospitalization in the past 6 months, a hospitalization in the past 45 days; history of CHF or an asthma; and total Medicare charges in the past 3 months and the past 12 months. 

The excess cost of pneumonia was then estimated from a multivariate generalized linear regression of total Medicare allowable charges on significant clinical and socio-demographic factors using a log-link function and a gamma distribution appropriate for modeling health care costs with a heavily skewed right tail[26–28]. The regression model was applied after the matching of cases and controls to additionally adjust for potential, observable confounders between cases and controls in the estimated marginal mean costs[29]. This model included most of the variables in the propensity model, with the addition of CMS region and the log of Medicare-allowable charges in the past 12 months. The excess direct medical cost of pneumonia was estimated as the difference between the marginal mean cost for cases and controls. Excess pneumonia costs were estimated for the quarter during which the index hospitalization or the matched control was identified and for each subsequent quarter over the two-year period inclusive of all persons alive at the start of each quarter. 

Additionally, to examine the potential impact of pneumonia on COPD severity, a sub-analysis on the rates of home oxygen utilization before and after hospitalization for pneumonia was performed. All medical costs were estimated using actual dollars for the year of expenditure and subsequently adjusted to 2010 constant dollars using the medical component of the Consumer Price Index. 

The study was approved by the Brandeis University Institutional Review Board (IRB) and its HIPAA board and executed under a Data Use Agreement approved by the Centers for Medicare & Medicaid Services.

## Results

### Population Characteristics

The Medicare population in this 5% CCW sample included 1,565,638 beneficiaries. The prevalence rate of COPD was 15.7 percent in 2004. Persons with COPD were slightly older and less likely to be female, twice as likely to have Medicare eligibility based upon disability or ESRD, and more likely to live in areas where median household incomes were in the lowest quartile. The prevalence rates of diabetes, CHF, ischemic heart disease, and atrial fibrillation were higher in elders with COPD than in those without COPD (Table **1**). 

**Table 1:** Selected characteristics of elder Medicare beneficiaries by COPD status in 2004*.

	**No COPD**		**COPD**		**COPD/CHF**
**Number of beneficiaries**	1,319,680		245,958		118,617
As % of all beneficiaries	84.30%		15.70%		7.60%
**Personal Characteristics**					
**Females, %**	59.70%		54.50%		55.10%
**Age (yrs), mean (sd)**	75.8 (7.3)		77.4 (7.2)		78.9 (7.4)
**Medicare-Disabled/ESRD, ** **%**	7%		14%		16%
**Bottom quartile household income,** **%**	24%		30%		31%
**Comorbidities**					
**Diabetes**	21.00%		33.60%		44.70%
**CHF**	17.80%		48.20%		100.00%
**Ischemic heart disease**	35.50%		65.40%		84.60%
** Atrial fibrillation**	9.60%		20.60%		36.00%

* COPD consists of patients with an ICD-9 code for COPD regardless of presence or absence of CHF. COPD/CHF consists of patients with ICD-9 codes for both diagnoses.

### Incidence of Pneumonia

Table **2** shows the crude incidence rates of pneumonia by category, COPD status, and concomitant COPD/CHF during the three year study period, 2005-2007. A six-fold difference in the crude incidence of any pneumonia was observed between elders with COPD and those without COPD ( 167.6 [CI, 166.7-168.5] per 1,000 person-years compared with 29.5 [CI, 29.3-29.6] per 1,000 person-years, respectively (RR = 5.7, p<0.01). An eight-fold difference in the incidence of pneumonia was observed among elders with COPD/CHF (240.2 [CI, 238.7-241.8] per 1,000 person-years, [RR = 8.1, p<0.01]) compared against elders without COPD. Specifically for inpatient primary pneumonia, elders with COPD had incidence rates nearly eight times that of elders without COPD (54.2 [CI, 53.6-54.7]/1,000 person-years vs. 7.0 [CI, 6.9-7.1]/1,000 person-years; RR=7.7, p<0.01 ). 

**Table 2:** Incidence of pneumonia by COPD status (per 1000 person-years)*.

		**COPD status**				
		**No COPD**	**COPD**	**IRR of COPD vs. No COPD**	**COPD/CHF**	IRR* of COPD/CHF vs. No COPD
Outpatient pneumonia only		15.1	64.7	4.3	86.2	5.7
	CI	15.0-15.3	64.1-65.3		85.3-87.2	
Inpatient primary pneumonia		7	54.2	7.7	77.2	11.0
	CI	6.9-7.1	53.6-54.7		76.3-78.1	
Any pneumonia+		29.5	167.6	5.7	240.2	8.1
	CI	29.3-29.6	166.7-168.5		238.7-241.8	

* IRR denotes incidence risk ratio. COPD consists of patients with an ICD-9 code for COPD regardless of presence or absence of CHF. COPD/CHF consists of patients with ICD-9 codes for both diagnoses.

+ Inclusive of all cases of pneumonia, i.e., outpatient pneumonia, inpatient primary pneumonia and inpatient secondary pneumonia

Note: all relative risk ratios are significant at p<0.01

### Excess Direct Medical Costs of Inpatient Primary Pneumonia

**Figure 1:**
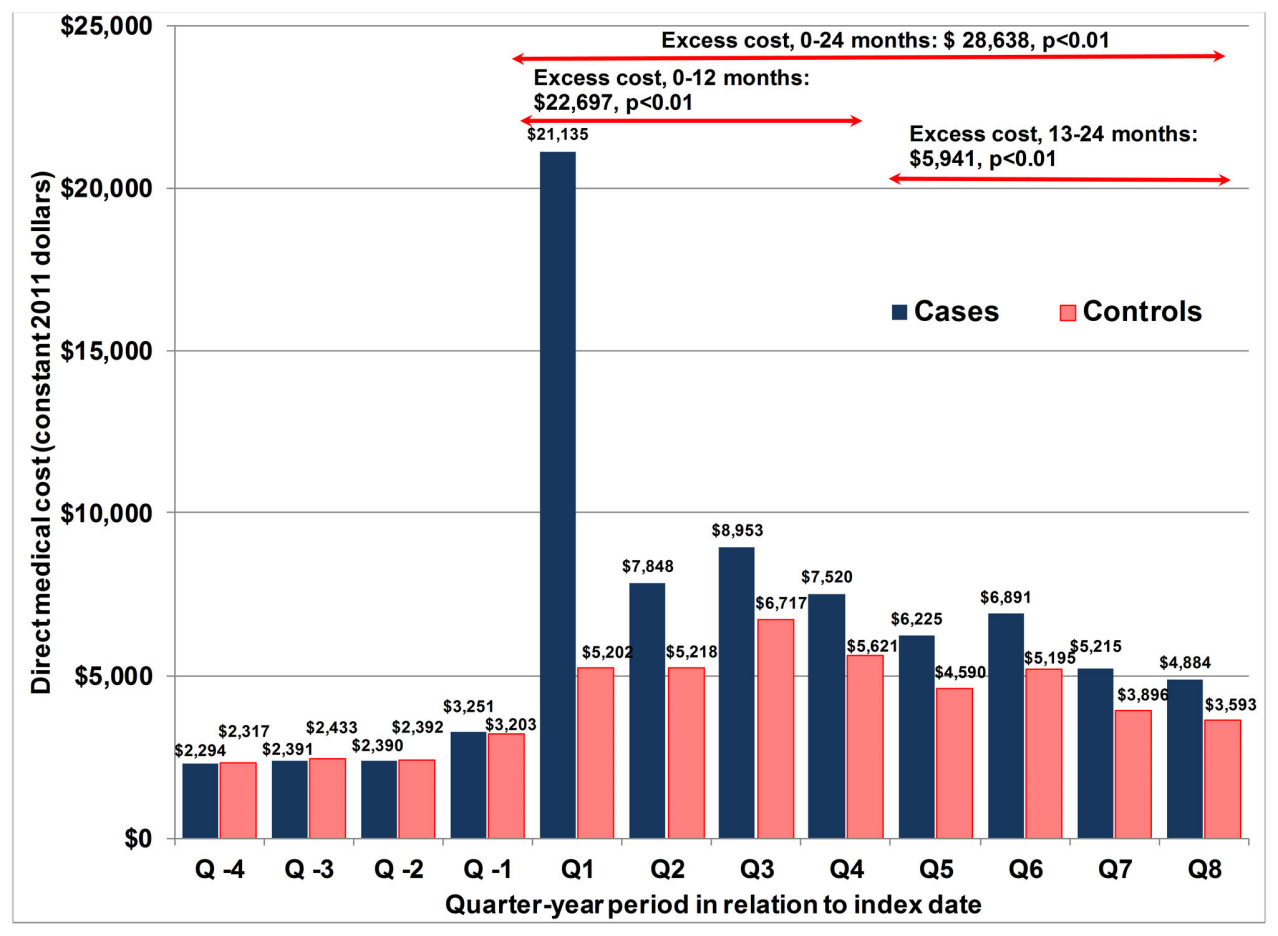
Quarterly direct medical cost and excess medical cost of 2005 inpatient primary pneumonia cases and controls in Medicare beneficiaries with COPD. Q1 corresponds to the quarter in which index inpatient primary pneumonia occurred. There are four quarters before index pneumonia episode (Q-1 to Q-4) and eight quarters following it (Q1 to Q8). Inpatient pneumonia is based on ICD9 coded claims with pneumonia as the principal discharge diagnosis. Excess direct medical cost estimates are based on 9,984 matched pairs of cases (inpatient primary pneumonia) and controls among COPD patients and subsequent multivariate generalized linear regression using a log-link function and a gamma distribution. Costs estimates are in 2010 constant dollars.

Figure **1** compares the quarterly costs of 9,984 cases (persons with inpatient primary pneumonia) and 9,984 controls (comparable persons without inpatient primary pneumonia) among elders with COPD starting one year prior to the index primary pneumonia hospitalization (and for two years following the pneumonia episode). Cases and controls had similar baseline costs, comorbidities, and demographics indicating successful matching on key characteristics (data not shown). Cases had an average cost of $ 45,456 from the quarter with the index pneumonia through the end of the first year. By contrast, controls had an average cost of $ 22,759 during the same period of time. Therefore, cases had an excess cost of $ 22,697 (p<0.01) during the first year following an inpatient primary pneumonia. Most of the first-year excess cost occurred during the first quarter (70.2%). During the second year, the excess cost of cases was $ 5,941 ($23,215 in cases vs. $ 17,274 in controls, p<0.01).

Lastly, our analysis on oxygen utilization revealed that a higher proportion of elders was receiving oxygen therapy following hospitalization for a primary pneumonia as compared with prior to the hospitalization. Prior to the index hospitalization 20.9 percent of elders with COPD had outpatient claims for receipt of oxygen, whereas the quarter following the quarter of the pneumonia hospitalization 31.6 percent were receiving oxygen therapy. 

## Discussion

Pneumonia, an identified cause of COPD exacerbations, was found to occur much more frequently among elders with COPD compared with those who do not have COPD. And this differential increased among elders with COPD/CHF. The risk for inpatient primary pneumonia was nearly eight times greater in elders with COPD and eleven times greater in elders with coexistent COPD/CHF compared with elders without COPD. 

Consistent with other studies, our study on nearly 1.6 million Medicare elders found that elders with COPD had higher rates of chronic conditions[2,6,11,30–32]. This increase in comorbidity partially explains the higher incidence of pneumonia. In 2004, 33.6 percent of elders with COPD had diabetes compared with 21 percent of elders without COPD; 48.2 percent of beneficiaries with COPD/CHF and 65.4 percent had ischemic heart disease (IHD). Additionally, elders with COPD were more likely to have Medicare eligibility based upon disability or ESRD, and more likely to live in areas where median household incomes were within the lowest quartile. 

The higher incidence of pneumonia in COPD patients may be explained by the lung changes associated with COPD (hypersecretion, bacterial colonization, impaired immunity) that lower an individual’s resistance to contracting pneumonia and make one prone to recurrent cycles of lung infection, further lung damage and to systemic effects that exacerbate other chronic conditions or impaired immune mechanisms[1,33–35]. And, as a result of the synergistic impact of COPD/CHF, recent research has highlighted the need to evaluate elders with COPD for CHF and test for COPD in the presence of CHF to minimize acute exacerbations and optimize treatment[12,13,15]. 

The estimated marginal mean cost difference between elders with COPD and inpatient primary pneumonia (cases) compared with matched elders with COPD and no pneumonia (controls) remained elevated through two years from the index pneumonia hospitalization. While the majority of excess direct medical costs occurred during the quarter of the index hospitalization, cost differences remained throughout the two years of follow-up. At the end of year one, the estimated total excess cost of cases was $22,697. During the second year, the excess cost of cases was $5,941. About one quarter (26.2%) of the excess direct medical cost occurred after the initial quarter of the index pneumonia. It must be noted that more cases died over the two-year period compared with controls. The costs for both cases and controls increased after the matched index hospital admission for pneumonia in cases. This may be explained by the progression of COPD over a two-year period among elders surviving with COPD.

To test a working hypothesis that inpatient primary pneumonia in elders with COPD results in further progression of COPD a sub-analysis was conducted on oxygen therapy utilization prior to and following the hospitalization. Claims data analysis do not yet permit verification of COPD staging as defined by the American Thoracic Society, the British Thoracic Society, or the GOLD criteria[36,37]. However, these guidelines for the staging and treatment of COPD recognize chronic hypoxemia as an indicator of advanced COPD – stage 3 or 4[38–41]. Medicare funds oxygen therapy only in cases of chronic hypoxemia defined specifically as a PaO_2_ of < 55 mm Hg[42]. Evidence of oxygen therapy has been employed by researchers as an indicator of disease severity as it is observable in claims and hypoxemia does denotes progression of COPD [43,44]. Our finding of increased oxygen utilization following hospitalization for pneumonia supports the current medical opinion that pneumonia infection results in permanent damage to the airways and further progression of COPD[35,45,46]. This finding may also partially explain the lingering financial impact of pneumonia infection in an elderly cohort with underlying COPD as oxygen therapy is a significant cost in the treatment of COPD[47–49]. 

The excess cost of pneumonia in elders with COPD has significance for national health spending. Given an estimated COPD prevalence rate of 15.7 percent and 29.3 million aged Medicare fee-for-service beneficiaries in 2010, 4.6 million Medicare elders have COPD. Applying our incidence of inpatient primary pneumonia in elders with COPD (54.2/1,000) we estimate almost 250,000 annual hospitalizations, with excess direct medical costs exceeding $5.7 billion during the first year following the pneumonia hospitalization. This estimate includes only fee-for-service beneficiaries (approximately 80% of elderly Medicare beneficiaries[50]) and does not include incidence and cost of inpatient pneumonia as a secondary diagnosis in hospital discharges. Pneumonia as a secondary diagnosis in hospitalizations suggests hospital acquired pneumonia, which was not a subject of this investigation.

The observed excess direct medical cost of inpatient primary pneumonia in elders with COPD during the first quarter was comparable to that observed in a prior study of pneumonia in elders overall without specifically analyzing those with COPD ($15,933 versus $13,632)[18]. However, the total excess direct medical cost in elders with COPD and inpatient primary pneumonia is much higher than in all elders over the two-year follow-up period providing support for the theory that pneumonia infection in COPD may result in permanent progression of the disease process. 

### Limitations

The analysis was based exclusively on administrative claims data. While our claims algorithm has been previously validated, claims data may miss some pneumonia episodes as claims are submitted for payment, not research. Additionally, the estimated pneumonia incidence rates are crude rates unadjusted for potential confounders but stratified by no COPD, COPD, and COPD/CHF. Moreover, given the underdiagnosis of COPD[51–54] estimates of crude incidence rates and relative risk ratios are likely to be biased downward. Our direct medical cost calculations excluded pharmacy expenditures as pharmaceutical claims data was not available. Prescription medications, particularly long-acting beta agonists and inhaled corticosteroids, constitute an important component of health care cost in the treatment of COPD [4,55–58]. 

Although we used propensity score matching and direct matching on key variables, our method balances only factors observable in claims data. The cases and controls could be well balanced within each stratum of propensity scores and differ on other important clinical markers not observed in the claims data. Nevertheless, our covariate for the bottom quartile household income served as a proxy for some lifestyle factors associated with income.

## Conclusions

This study has implications for health policy. COPD and pneumonia are high cost conditions individually and concomitantly, pneumonia and COPD increase health care cost synergistically in the elderly. Therapies aimed at preventing exacerbations in general and pneumonia in particular have the potential to reduce morbidity, improve quality of life and reduce costs for Medicare beneficiaries with COPD[4,59]. As elders with COPD are at increased risk of acquiring pneumonia (an important source of COPD exacerbation) and suffering both short and long-term health consequences, preventing pneumonia may have a beneficial impact on utilization and cost. 

## Supporting Information

File S1 **Methods: Chronic Condition Warehouse (CCW).**
(DOCX)
