# Supplementary material for: Incidence and Cost of Pneumonia in Older Adults with COPD in the United States
Source: PLoS One. 2013 Oct 9;8(10):e75887. doi: 10.1371/journal.pone.0075887 (PMC3794002; doi:10.1371/journal.pone.0075887)
Supplement: File S1 — Methods: Chronic Condition Warehouse (CCW). (DOCX) [file pone.0075887.s001.docx]

**On-Line Supplement to:**

***Incidence and cost of pneumonia in older adults with COPD in the United States***

**Methods: Chronic Condition Warehouse (CCW)**

In this study we created a person-level analytical file with necessary data linkages across the years by requesting research identifiable files (RIF), through a data use agreement (DUA) with CMS.  To ensure the protection of beneficiaries’ privacy, the DUA was reviewed by the CMS Privacy Board, the data were maintained securely with access limited to authorized persons, and the study was approved by the Brandeis University Institutional Review Board (IRB) and its HIPAA board as well as the Centers for Medicare & Medicaid Services. As the investigators had no contact with beneficiaries, did not have their names, addresses, or Social Security numbers, and report only aggregate information, the IRB agreed that informed consent from study participants was not necessary.

The CCW includes Parts A and B administrative claims data for hospital, outpatient, and skilled nursing facility claims, plus demographic and eligibility information. Data on medication use and costs were not available at the time of the study.

Classification of COPD Status:

The CCW summary file contains chronic disease specific ‘flags’ in accordance with specified algorithms in the claims data files. The date of first observation is included with the disease specific ‘flag.’ A COPD ‘flag’ is generated if any of the following ICD-9 codes (491.0-2, 491.8,9, 492.0, 492.8, 494.0, 494.1, and 496) are observed during a one-year period in at least one inpatient, skilled nursing home care, or home health agency claim, or two Part B claims, including any combination of claims at least one day apart. [http://www.resdac.umn.edu/CCW/data_available.asp#1]

Identification of congestive heart failure (CHF):

Within individuals with observed COPD using the CCW specified algorithm, the coexistence of congestive heart failure was identified by the CCW heart failure ‘flag.’ The heart failure ‘flag’ is generated if any of the following ICD-9 codes (398.91, 402.1, 402.11, 402.91, 404.01, 404.11, 404.91, 404.03, 404.13, 404.93, 428.0, 428.1, 428.20, 428.21-.23, 428.30-.33, 428.40-.43, 428.9) are observed during a one-year period in at least one inpatient, skilled nursing home care, home health agency claim, or Part B claims.

[http://www.resdac.umn.edu/CCW/data_available.asp#1]
